# Book Reviews

**Published:** 1894-02

**Authors:** 


### *BOOK REVIEWS.*

CLINICAL GYNECOLOGY. A HAND BOOK OF DISEASES PECULIAR TO WOMEN. By Thomas More Madden, M. D., F. R. C. S., Ed. J. B. Lippincott Company. Philadelphia, 1893.

This work is intended to give the practical experience collected by Dr. Madden from years of active practice and is a revised compilation of his lectures and journal contribution upon clinical gynecology.

The work is well adapted to the purpose for which it was intended, giving to the student and busy practitioner those methods found by long years of experience best suited in the hands of so competent a man as Dr. Madden. When deemed advisable, reference to other works is made, giving the views of their authors. Dr. Madden does not rely upon the authority of German and French gynecologists, as many of our present day writers and teachers are wont to. He says :

"Should we, however, need, as we all sometimes do, further light to guide our steps in the often dark and difficult parts of gynecology, it is not to France or Germany but to America that we may best turn for guidance. Had we no other reason than this for gratitude to our transatlantic brethren it should be sufficient for us to recall the memory of the first ovariotomist, who, in a remote Kentucky village, alone and under the most unfavorable circumstances, successfully accomplished what the leaders of surgery in the great centers of European science, from Ambrose Pare to Hunter and Lister, had long previously dreamt of but had not ventured to attempt, and so inscribed the name of Ephraim McDowell in imperishable characters on the annals of medicine and humanity.

A PRACTICAL TREATISE ON MATERIA MEDICA AND THERAPEUTICS. By Roberts Barthlow, M. A., M. D., LL. D., Professor of Materia Medica, General Therapeutics and Hygiene in Jefferson Medical College of Philadelphia; Fellow of the College of Physicians; Member of the American Philosophical Society; Honorary Fellow of the Royal Medical Society of Edinburg; Honorary Member of the Société Medico-pratique de Paris and of various national, state and county medical societies. Author of a Treatise on Practice of Medicine; of a Treatise on Medical Electricity; of a Manual on Hydropathic Medication of the Russell and Juett Prize Essays, etc. Eighth edition, revised and enlarged.

We have just received from D. Appleton & Co. the latest edition of "Barthlow's Materia Medica and Therapeutics." It has long been before the public and hardly needs an introduction. But we find new and valuable medicines and remedies described. Also the most valuable of the many proprietary medicines which are not found in the Pharmacopœia. We are also glad to see the metric system used, for it is only a question of time when all physicians will use it, as it is the only rational system of weights and measures. For, founded on a decimal system as it is, we are much less liable to make mistakes than with the old system of grains, drachms, etc. We heartily recommend this edition to all students and physicians who wish to keep up with the times. J. L. C.

---

HERNIA: ITS PALLIATIVE AND RADICAL TREATMENT. By Thomas H. Manley, M. D., Visiting Surgeon the Harlem Hospital, New York, Member National Association of Railway Surgeons, etc. Medical Press Company, 1725 Arch street, Philadelphia.

This work is the outcome of much study and experience in the subject of Hernia, and Dr. Manley's discussion of it in all its aspects is both able and scientific. The first part of the work relates to the hernia of infancy and childhood, and the proper curative means are described, non-operative and operative.

Hernia in the adult is treated very fully, especially the radical cure of the reducible and irreducible forms by surgical methods. The history and present status of radical operations are exhaustively discussed. The author advocates the use of cocaine (one per cent. solution) in operating for strangulated hernia, and describes in detail the technique of its administration. Dr. Manley's book is an excellent contribution to the literature of Hernia, and will be found of great practical service.

The typography is not well executed, and the absence of a table of contents is a disadvantage.

